# Phimosal Paraplegia

**Published:** 1875-10

**Authors:** C. E. Beardsley


Phimosal Paraplegia.—*By C. E. Beardsley, M.D.*—In 1865, January 18th, a boy, fourteen years of age, was brought to me for examination and treatment, by a widowed mother, whose husband had been killed on the battle-field of Stone river.

The lad was tall, with dark eyes and hair, also extremely emaciated; he had no power over the lower extremities, and, seemingly, but little strength in the vertebral column, as he was unable to hold his head erect; he was also a great sufferer from spasms, epileptiform in character. Simply pronouncing the word spasm or fit in his presence would throw him into convulsions, or the least shock, as concussion of the hands, would produce the same result. The convulsions could be produced in the same manner as often as he would arouse from the stupor which followed each attack.

The history of the case, as gathered from the mother, was of but little value. She, like most women of inferior intellect, was extremely modest—too much so to lead us to the real cause. Like the woman of old, she had spent all she had on doctors, without benefit, but the patient rather grew worse.

I undressed the boy, better to observe his movements and to examine him for some lesion, I knew not what. The mother said he was thrown from a horse a year before the occurrence of the spasms; and, naturally enough, she attributed all the present trouble to that fall. The boy, now, after two years of a life of suffering, is semi-idiotic, with complete paralysis of the lower extremities. While he was in the nude state and in one of the convulsions, I observed that there was a partial erection of the penis, with dribbling of urine, which induced me to examine the parts, and if possible, the bladder, for stone, as the most probable cause of the trouble, by a reflected irritability. On attempting to introduce a sound into the bladder, I was foiled by a very small meatus and an inability to retract the prepuce. I further discovered, just behind the corona glandis and beneath the integument, what seemed to be a ring of some hard material. On a second attempt to retract the foreskin, I discovered that it too was adherent to the gland, with slight balanitis. I therefore circumcised the lad, tearing back the mucous membrane from off the gland, turning out at the same time a ring of sebaceous material the density of an ordinary cheese crust. I used water dressings to the parts, and gave him internally iodide of potassa, and valerian in small doses, three times per day, with liberal diet. He was to have this treatment for the time being only, I expecting to find a stone in the bladder as soon as the patient would recover from the circumcision sufficiently to allow introduction of a sound; but the boy gained in strength from day to day, the spasms and all untoward symptoms suddenly disappeared, and by the time I again saw him (six weeks) he had regained the use of his limbs as to be able to walk with some assistance, his mind becoming clearer as he gained strength, until complete recovery. I gave him the potash and valerian but one week, and no other medicine at that time or afterwards.

The young man is living now in my neighborhood, a perfect specimen of health and strength.—*Med. and Surg. Reporter.*

